# Bioinspired Magnetic Nanochains for Medicine

**DOI:** 10.3390/pharmaceutics13081262

**Published:** 2021-08-16

**Authors:** Slavko Kralj, Silvia Marchesan

**Affiliations:** 1 Department for Materials Synthesis, Jožef Stefan Institute, Jamova 39, 1000 Ljubljana, Slovenia; 2 Department of Pharmaceutical Technology, Faculty of Pharmacy, University of Ljubljana, Aškerčeva 7, 1000 Ljubljana, Slovenia; 3 Department of Chemical and Pharmaceutical Sciences, University of Trieste, 34127 Trieste, Italy; smarchesan@units.it

**Keywords:** biomineralization, biocompass, magnetotactic bacteria, magnetosome chains, magnetic navigation, magnetic assembly, superparamagnetic iron oxide nanoparticles, single domain particles, magnetite, magnetoreception

## Abstract

Superparamagnetic iron oxide nanoparticles (SPIONs) have been widely used for medicine, both in therapy and diagnosis. Their guided assembly into anisotropic structures, such as nanochains, has recently opened new research avenues; for instance, targeted drug delivery. Interestingly, magnetic nanochains do occur in nature, and they are thought to be involved in the navigation and geographic orientation of a variety of animals and bacteria, although many open questions on their formation and functioning remain. In this review, we will analyze what is known about the natural formation of magnetic nanochains, as well as the synthetic protocols to produce them in the laboratory, to conclude with an overview of medical applications and an outlook on future opportunities in this exciting research field.

## 1. Introduction

**Figure 1:**
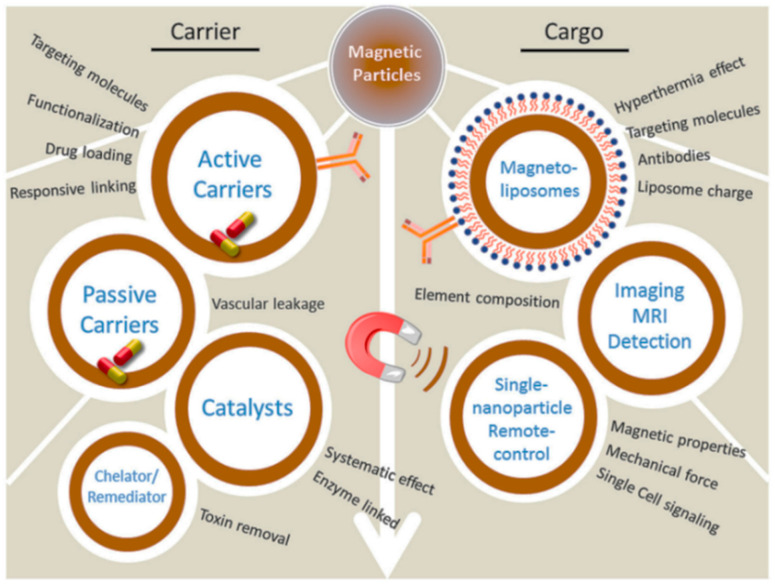
Biomedical applications for magnetic nanoparticles. MRI = magnetic resonance imaging. Reproduced from [2]. CC BY 4.0 license.

Superparamagnetic iron oxide nanoparticles (SPIONs) are a topic for vibrant scientific research, and also for applications in medicine (Figure 1) [1,2]. They typically display a spherical morphology and have been envisaged for numerous biomedical uses, including therapy and diagnostics [3,4]. In particular, SPIONs are clinically used as contrast agents for magnetic resonance imaging (MRI) and hyperthermia, which consist of nanoparticles producing heat upon exposure to the high frequency alternating magnetic field [5]. Furthermore, novel nanoparticle designs opened new avenues for magnetically guidable drug-delivery systems, towards selective targeting of organs or tissues, with the assistance of an external magnetic field [6]. Importantly, SPIONs are recognized as safe for human use by the main regulatory agencies [7].

Their biocompatibility and low toxicity have also emerged in clinical trials. For instance, a phase II study reported their promising performance to treat recurrent glioblastoma through intratumoral thermotherapy in conjunction with fractionated stereotactic radiotherapy [8]. The adverse effects of the proposed therapeutic approach were moderate, with no occurrence of serious complications. The thermotherapy used magnetic nanoparticles, allowing for a reduced radiation dose of the combined radiotherapy, and the approach proved to be safe and effective. Importantly, the overall survival from the diagnosis of the first tumor recurrence was longer, relative to the conventional therapies commonly used to treat recurrent glioblastoma. Indeed, nanomaterials are well-known for their potential to make a qualitative leap for the development of effective and innovative solutions in both therapy and diagnosis [9].

The safety aspect is further supported by the fact that certain animal species produce magnetic nanoparticles themselves [10]. This process occurs naturally in sensory organs, and the magnetic nanoparticles are hypothesized to allow for navigation and geographic orientation of migratory birds, honeybees, pigeons, fruit flies, salmon, etc. The sensory mechanism most likely involves complex mechano- and/or magneto-receptors that are sensitive to extremely weak variations in the mechanical forces produced by spatially aligned chains of magnetic nanoparticles. However, the exact mechanism of magnetoreception in animals is still subject to debate, because the corresponding biochemical components linked to the sensory receptors have yet to be identified, isolated, and properly analyzed [11]. Currently, there is another hypothesis for animal sensing the Earth’s magnetic field that includes cryptochrome *Er*CRY4 protein, which has recently been found in the eyes of migratory European robins, and which possesses the right physical properties to be a magnetosensor [12,13,14]. The cryptochrome protein absorbs light and gets photoexcited, meaning that magnetically sensitive intermediates, known as radical pairs, are generated [15]. Lu et al. have recently shown that *Er*CRY4 protein has the ability to form long-lived radical pairs that have high magnetic sensitivity and can fulfill the physical requirements needed for magnet sensing [12]. In particular, site-specific mutations of *Er*CRY4 revealed the roles of four flavin–tryptophan radical pairs in generating magnetic-field effects, and in stabilizing potential signaling states in a way that could enable sensing and signaling functions. Notably, these two proposed sensing mechanisms might be coherent and, indeed, mutually present in some migratory animals [10,15].

There are three navigational phases that some migratory animals exploit for their navigation. They are (1) long-distance phase, (2) homing phase, and (3) pinpointing the goal phase [15]. Therefore, it is highly unlikely that a single sense or cue is used exclusively during the animal journey. However, an unanswered question pertains: what determines the animals’ switching from one navigational phase to the other, and how do processing strategies in the nervous system take place between phases [15]? The focus of this review will be directed to the discussion of the magnetic nanoparticle-based hypothesis, and not of the radial-pair-based magnetoreception theory.

The geomagnetic field magnitude at Earth’s surface of ca. 0.3 to 0.6 Gauss is large enough to produce magnetic torque of chain-like magnetic particles suitable to orient the particle chain in a direction towards the Earth’s magnetic pole [16]. The magnetic force that is exerted on individual superparamagnetic iron oxide nanoparticles is not sufficient to spatially guide the nanoparticle in a liquid, even with their exposure to a magnetic field that is orders of magnitude larger than the geomagnetic one. Therefore, the chain-like alignment of the individual superparamagnetic nanocrystals cannot be achieved exclusively using the Earth’s magnetic field, due to the fact that the magnetic dipole interactions of such small nanocrystals are too weak, and they are overcome by the random nature of thermal fluctuations at ambient temperature.

Interestingly, millions of years of evolution have created a solution in the formation of single-domain ferrimagnetic nanoparticles with sizes between 35 and 120 nm [17]. Practically, such nanoparticles behave like tiny permanent magnets that spontaneously assemble in rigid chain-like structures (i.e., nanochains), thanks to attractive, magnetic dipole–dipole interactions, without the assistance of an external magnetic field. The nanochains of ferrimagnetic nanoparticles are very sensitive to extremely weak magnetic fields, and therefore can produce sufficient mechanical torque on accompanying mechano- and/or magneto-reception in living organisms in a field as weak as the geomagnetic field.

These ferrimagnetic nanochains can be found in prokaryotic microorganisms, such as magnetotactic bacteria as first described by Salvatore Bellini in 1963 [18]. The first report on iron biomineralization in magnetotactic bacteria was given by Blakemore half a century ago [19]. These bacteria can be magnetically isolated and the existence of their ferrimagnetic magnetite particles in the so-called “magnetosomes” was confirmed by different techniques, including transmission electron microscopy (TEM).

**Figure 2:**
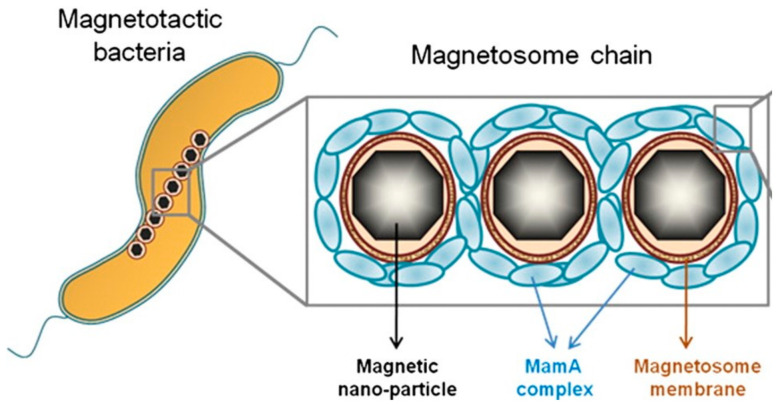
Schematic presentation of magnetotactic bacterium with a magnetosome chain. The magnetosome consists of lipid invaginations, each one enclosing a ferrimagnetic nanocrystal. The 1D chain of magnetosomes is decorated by MamA homo-oligomers. Reproduced from [24]. Copyright 2011, with permission from PNAS.

Magnetosomes are biological vesicles with ferrimagnetic nanocrystals that are enclosed within a lipid-bilayer membrane, i.e., the magnetosome membrane (Figure 2) [20,21,22]. The magnetosome is unusual in protein composition compared to other intracellular vesicles in prokaryotes, because it exhibits proteins that are unique to the magnetosome membrane. The proteins are classified based on their location on the magnetosome membrane (Mam), or specific to the magnetic particle membrane (Mms), and they are encoded by the corresponding *mam* and *mms* genes [17], respectively. Furthermore, the chains of ferrimagnetic magnetosomes create their own local magnetic field and probably interact with adjacent magnetotactic bacteria through magnetic interactions. Although such ferrimagnetic nanochains can be harvested from the magnetotactic bacteria in the laboratory, they cannot be easily prepared in stable colloidal suspension, due to their ferrimagnetic properties. The ferrimagnetic nanochains could agglomerate irreversibly, due to attractive magnetic interactions, when they get in close proximity with each other. This problem significantly limits their potential use in many biomedical applications, although their anisotropic shape and the possibility they offer for an easy spatial magnetic guidance, are both highly desired and advantageous properties of magnetic materials.

In this review, we discuss the formation of ferrimagnetic nanochains in magnetotactic bacteria as a naturally driven biomineralization process (Section 2.1) [23]. The aim of this review is to demonstrate diverse bioinspired approaches for the synthesis of magnetic nanochains with optimal properties for biomedical applications (Section 2.2). Furthermore, the applications of magnetic nanochains in biomedicine are discussed (Section 3). Finally, we conclude the review with our view on the future progress of magnetic nanochains in biomedicine (Section 4).

## 2. Natural and Bioinspired Synthetic Approaches to Form Magnetic Nanochains

Many living species are supposed to produce iron oxide-based nanoparticles, as well as other minerals, in a process that is generally termed biomineralization. It involves complex cellular machinery, including the genetic code regulating the particles’ crystallinity and growth, as well as their spatial organization at the nanoscale. Frequently, such biological nanoarchitectures possess unique chemical structures and compositions, both of which can hardly be replicated in the lab. Therefore, a better understanding of the mechanisms involved in the biomineralization process can guide future biomimetic designs of bioinspired magnetic nanomaterials with outstanding magnetic properties. In this regard, the use of advanced characterization techniques is needed to better understand the mechanisms involved in the biomineralization process, as well as to pinpoint all the steps involved in the biosynthetic pathway. The most typically used analytical methods are listed in Table 1.

### 2.1. Biomineralization and Magnetosome Chain Formation in Magnetotactic Bacteria

There are many living species with the ability to produce pure inorganic or composite nanocrystalline biomaterials that include mostly oxides, hydroxides, phosphates, sulfates and carbonates, to mention a few. The broader use of the term biomineralization addresses the questions pertaining to how the organisms assemble such nanocrystals, which biological determinants are involved in the hierarchical organization of the precipitated biomaterials, and what are the biominerals’ biological roles and functions. Therefore, biomineralization is a very interdisciplinary field that combines the expertise of biologists, chemists, geologists, materials scientists, and engineers, among others [44].

Understanding the mechanisms involved in the versatile biomineralization processes is of special interest, because organisms have the ability to produce stable minerals in a polymorph form that does not correspond to what is expected by classical thermodynamic rules [45]. Furthermore, organisms are able to adjust the crystallinity and crystal shape to meet their biological needs, contrary to the basic rules of crystallographic symmetry and relevant laws. A typical example is offered by magnetotactic bacteria that form elongated spinel nanocrystals, which are very uncommon because the spinel is known to crystallize in a cubic system, for which crystals with anisotropic shapes are not expected [46]. Such phenomena are of inspiration for scientists and may offer creative solutions for new materials’ design principles. In this section, we focus on intracellular mineralization, which allows for the highest degree of control over the nanochains, and it is typical of microorganisms [47]. Since this review is focused on bioinspired magnetic nanomaterials, we will limit the topic to bacteria capable of forming iron oxide minerals (Figure 2). Moreover, magnetotactic bacteria are able to spatially align magnetic nanoparticles in a chain-like formation that significantly attracts our interest. Further details can be found in a comprehensive review on the biomineralization of iron oxides in magnetotactic bacteria and other organisms that have recently been described by Faivre and Ukmar Godec [48].

In general, mineralization, either chemical or biological, should follow basic nucleation rules. The crystal formation requires a confined space with a supersaturated concentration of a solute. Therefore, the critical nucleus can be formed when the concentration of the dissolved species exceeds the equilibrium solubility. However, the so-called non-classical biomineral formation pathways have also been proposed in organisms where re-crystallization from poorly crystalline precursors takes place [49,50,51]. Single-cell organisms usually uptake elements from the environment through active or passive pathways, and these elements are either ions or complexes. Then, once elements are internalized and reach a suitable concentration, the nucleation appears through the fine regulation offered by complex cellular machinery.

**Figure 3:**
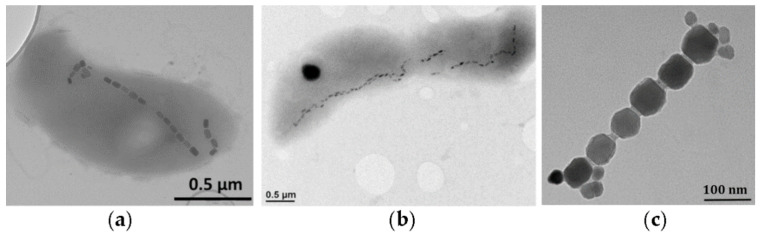
Transmission electron microscopy (TEM) images of magnetotactic bacteria with 1D chains of magnetosome nanoparticles of different morphologies. (**a**) *Magnetovibrio blakemorei* strain MV-1 where elongated prismatic nanocrystals can be found, (**b**) *Desulfovibrio magneticus* strain RS-1 which produces bullet-shaped nanoparticles, and (**c**) TEM images of magnetosome chain from a lysed cell of *Magnetospirillum magneticum*, strain AMB-1. Each cuboctahedral magnetite nanocrystal is surrounded by the phospholipid magnetosome membrane, which often remains stable even after cell lysis. Adapted from [17], Copyright 2013, with permission from Elsevier.

Magnetotactic bacteria are Gram-negative, they produce and assemble magnetic nanoparticles in magnetosome vesicles (Figure 3). Magnetic nanoparticles detected in magnetotactic bacteria are usually magnetite (Fe_3_O_4_), greigite (Fe_3_S_4_), or another, a less-oxidized form of magnetite, namely maghemite (γ-Fe_2_O_3_), thanks to the magnetosome membrane protecting magnetite particles from oxidation [52]. Interestingly, it is known that bulk magnetite possesses a larger value of magnetization per mass, compared to maghemite. The characteristics of the magnetosomes, such as size, number, and morphology are genetically controlled, and strain-specific [46]. The magnetite nanoparticles are consistent with three main crystal morphologies that are (1) cuboctahedral, (2) elongated prismatic, and (3) bullet-shaped (Figure 3) [17]. The size of the formed magnetic nanoparticles is usually between 35 nm and 120 nm, which means that nanoparticles are ferrimagnetic and single domain. Larger multi-domain iron oxide particles with size above approximately 200 nm possess reduced residual magnetization, compared to single-domain particles (with a size below approximately 100 nm), and such large multi-domain particles have never been identified in magnetotactic bacteria [17,53]. Through size selection of the magnetic nanoparticles, magnetotactic bacteria maximize the magnetic remanence per unit volume of material. Single-domain ferrimagnetic nanoparticles behave as tiny permanent magnets that easily align along the crystals’ axis of magnetization, so as to magnetically assemble the magnetosome particles in a chain-like formation [54]. Therefore, this property is at the basis for the formation of a “magnetic compass needle”, and hence it allows bacteria to spatially navigate in search of nutrients [55,56].

The biomineralization of the magnetic mineral phase in magnetotactic bacteria is controlled by the magnetosome membrane. The invagination of the cytoplasmic membrane is considered the initial step in the magnetosome biomineralization process [20]. The magnetite particles in magnetotactic bacteria are formed after the uptake of iron species, while oxygen originates from water. Iron is internalized as Fe^2+^ ions or Fe^3+^ complexes, and then it is transported intracellularly. Magnetotactic bacteria do not appear to possess a unique, iron-uptake machinery. There are ferric uptake regulator transcription factors that may play a role in the magnetosome biomineralization [57]. Remarkably, the iron content is greater than 3% of the overall dry matter, and this amount is several orders of magnitude larger than that of non-magnetic bacteria [58]. Once iron is taken up by cells, the phase transformation mechanism takes place and involves phosphate-rich iron (III) hydroxides, and iron (III) oxyhydroxide nanoparticles, as confirmed recently using advanced analytical techniques [59,60]. The presence of ferritin-like, and ferrihydrite-like intermediates was confirmed on the outer and inner sides of the magnetosome membrane, respectively, and it was shown that outer ferritin-like species are direct magnetite precursors [60]. The process of magnetosome formation is relatively rapid as demonstrated in vitro on *Magnetospirillum gryphiswaldense* [32]. Small and immature 5 to 10 nm-sized magnetite nanoparticles are formed simultaneously at multiple discrete sites along the bacterium in 30 min. Then, growing nanoparticles start to concentrate at mid-cell after 175 min and form initial chain-like assemblies. The process is complete within 6 h, at which point straight and tightly packed chains of mature nanoparticles are present at mid-cell.

**Figure 4:**
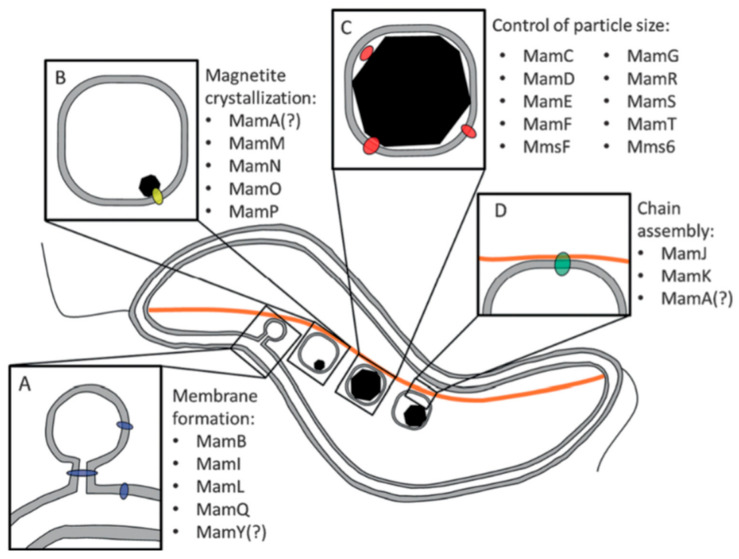
Proteins that are potentially involved in the different phases of magnetosome formation and assembly. (**A**) Magnetosome membrane formation; in particular, MamY (blue) could be used to shape and close the vesicle, and to sort further proteins. (**B**) Crystallization of magnetite, with MamO in yellow. (**C**) Particle size control. (**D**) Chain assembly, with MamJ (green) to anchor proteins, MamK filament proteins in orange, and potentially MamA. Reproduced with permission from [48], Copyright 2015, with permission from Wiley.

Magnetite nanoparticles’ biomineralization in magnetotactic bacteria has been postulated to be controlled by strain-specific proteins that are encoded by specific sets of genes, which are organized in the so-called chromosomal “magnetosome islands” (Figure 4) [61]. They encode a number of specific polypeptides and proteins, whose functions are still poorly understood. There are structural homologies among different sets of proteins responsible for certain functions in different bacteria strains. The amino acid sequences of these proteins display a high similarity to some metal transporters that are known to control the uptake of iron, as well as of some other metals. For instance, a common *mms6* gene encodes for the Mms6 protein, which is an amphiphilic protein located in the magnetosome membrane, and whose sequence is abundant with acidic amino acids at the C-terminus, and hydrophobic leucine and glycine amino acids at the N-terminus. This acidic protein assists with the iron-oxide nanoparticle formation, thanks to the known affinity between the iron atom and the carboxyl group. This observation was further supported by the fact that an *mms6* knockout mutant favored the synthesis of 50% smaller elongated particles, in contrast with the wild-type organism, where the cuboctahedral crystal morphology is dominant [62].

There are a few other acidic proteins similar to Mms6 such as MamC, MamD and MamG that are confined in the magnetosome membrane too [63]. Another acidic protein, MamJ, has been identified in *Magnetospirillum gryphiswaldense* where it plays a crucial role in the magnetosome formation, as well as in the magnetosome association with the cytoskeleton structure that aligns magnetite particles into magnetosome linear chains [32,64]. MamJ is a protein composed of 426 amino acids abundant with acidic glutamate residues, and a repetitive domain structure, both of which are characteristic of also other proteins that are involved in the biomineralization process [65]. The role of MamJ in the formation of functional magnetosomes was confirmed through the design, production and evaluation of MamJ-deficient (*ΔmamJ* cells) mutant strains [32]. The authors used a gene-deleted mutant to demonstrate that MamJ did not assist in the biomineralization process, because the magnetite particles were of identical size, morphology and number, relative to those in wild-type magnetosomes. However, mutant cells were unable to form straight magnetosome chains of magnetite nanoparticles. Instead, the nanoparticles were aggregated in compact, cluster-like deposits.

**Figure 5:**
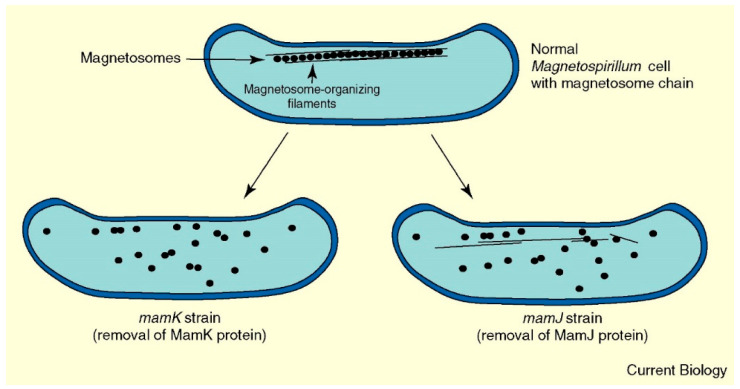
Cellular organization of bacterial magnetosomes. Reprinted from [70] copyright 2006, with permission from Elsevier.

Dunin-Borkowski et al. showed that linear chains of nanoparticles generate the highest possible magnetic moment for magnetotaxis, because the total magnetic dipole moment is the sum of the moments of individual single-domain ferrimagnetic particles [66]. Therefore, the chain-like assembly provides a means for the bacterium to spontaneously align with the geomagnetic field direction as it swims [67,68]. However, the chains of magnetic dipoles have an inherent tendency to agglomerate in order to lower their magnetostatic energy [69]. Therefore, the linear assemblies of ferrimagnetic magnetite particles should be stabilized sufficiently by organic matter, such as fibrillar cytoskeleton filament assemblies, in order to render the structure rigid enough to orient the whole bacterial cell in the direction of the external magnetic field (Figure 5).

Further analyses of magnetosomes isolated from *ΔmamJ* cells showed that the magnetosome membrane integrity was not affected by this mutation, since the magnetite nanoparticles from wild-type and mutant cells were both enclosed by an intact magnetosome membrane, while adjacent magnetite nanoparticles were densely linked with organic matter to form inter-particle junctions (Figure 5). Interestingly, when isolated, *ΔmamJ* cell-derived magnetite particles were no longer clustered, but spontaneously formed chains or flux-closed rings identical to those of wild-type cells [71,72]. Furthermore, the complete removal of organic matter from the surface of magnetite nanoparticles, by using sodium dodecyl sulfate, caused irreversible agglomeration of the particles. It was proposed that single-domain ferrimagnetic nanoparticles required a minimal thickness of the organic coating to significantly reduce the attractive magnetic dipole–dipole interactions among ferrimagnetic nanoparticles, due to particles’ steric repulsion. Therefore, in such a regime, the sterically stabilized ferrimagnetic nanoparticles could spontaneously form chains or other assemblies, because the dipole–dipole interactions had been sufficiently weakened, through larger distancing between adjacent nanoparticles. These findings suggested that the intact magnetosome membrane is required for particle alignment into the chain, while the ability to form chains in vitro is not affected by the deletion of MamJ.

Finally, the subcellular localization of magnetosomes in wild-type and mutant cells showed significant differences. Indeed, magnetosome chains in wild-type cells are usually positioned to the mid-cell, along the filaments that connect both cell poles in wild-type cells. Mature magnetosomes are in the middle of the chain, while empty vesicles and immature magnetosomes are placed at the chain terminus in wild-type cells. Conversely, *ΔmamJ* cells have their empty vesicles and immature magnetite nanoparticles distributed randomly in cytoplasm, with poor association with the filaments. These data elucidate the role of MamJ protein in the attachment of magnetosomes to the macromolecular cytoskeletal structure, hence in the formation of magnetosome chains.

However, MamJ is not the only protein involved in the chain-like assembly of magnetosomes. Another study showed that MamK is recognized as homologous to the bacterial actin-like MreB protein and it appears that MamK forms filaments in vivo [20]. In a mutant cell with a deletion of *mamK* gene, the magnetosomes were no longer organized in the chains, due to the interruption in the magnetosome–cytoskeleton association. The alignment and rotation of magnetotactic bacteria in the geomagnetic field are therefore enabled by mechanically stable MamJ–MamK protein interactions [25]. Magnetite chains in magnetotactic bacteria are mechanically highly stable since they remain unaffected by exposure to an external magnetic field of strength of 30 mT, which is about 500 times the strength of the Earth’s magnetic field. The magnetosome connector MamJ has been recognized as the weakest component of this assembly, although it can withstand an estimated magnetic force of 25 pN [25]. Indeed, it seems that magnetosome chain formation is a complex and highly dynamic process that involves (1) a finely regulated interplay of magnetic interactions among mature ferrimagnetic nanoparticles, (2) a physical contact between sterically stabilized adjacent nanoparticles, and (3) MamJ/MamK assistance to direct the assembly and localization of these organelles. Magnetosome assembly represents hence one of the highest structural levels achieved in prokaryotic cells [32].

### 2.2. Bioinspired Approaches for Magnetic Nanochain Synthesis

Inspired by the natural marvel of microbial mechanical and magnetic engineering of magnetotactic bacteria, researchers have been trying to understand and exploit these concepts to obtain novel nanoarchitectures with outstanding properties. The magnetic properties of the bioinspired chain-like nanostructures rely on the control over individual nanoparticle shape, size and crystal structure, as well as on the physical characteristics of their assemblies [73].

There are not many examples of syntheses of ferrimagnetic nanochains that mimic magnetosome chain-like assembly. This is due to the challenges that researchers face with a spontaneous magnetic agglomeration of ferrimagnetic nanoparticles. Recently, Zhang et al. showed the synthesis of ferrimagnetic magnetite nanocubes with an edge length of 25 nm [26]. These nanocubes were coated with a polymer to achieve steric hindrance, hence, to reduce the magnetic attractive forces between ferrimagnetic nanoparticles, due to larger inter-particle distance. However, the nanocubes spontaneously self-assembled into nanochains with random lengths. The length control of such ferrimagnetic chains is very difficult to achieve since this dipolar magnetic assembly is spontaneous and thus omnipresent [74].

Another bioinspired study recently presented by Sturm et al. showed similar ferrimagnetic magnetite nanochains that resembled the magnetosome chains found in magnetotactic bacteria [75]. Although the authors used a gelatin hydrogel to spatially separate the nanochains during their formation, it was not possible to exert control over the nanochains’ lengths within the hydrogel matrix. Finally, the formed ferrimagnetic nanochains displayed a strong tendency to magnetically collapse into irregular and irreversible aggregates, due to the uncontrolled assembly process. Therefore, researchers are actively seeking new strategies to obtain novel building blocks for magnetic nanochains with enhanced properties, and with the possibility to exert finer control over their length too.

#### 2.2.1. Synthesis of Superparamagnetic Magnetosome-Like Multicore Nanoparticle Clusters

Among the iron-oxide nanoparticles that are present in magnetotactic bacteria, only one type of single-crystal structure has been confirmed, namely magnetite, although it can occur in a variety of different crystal morphologies. These magnetite particles are single domain ferrimagnetic nanocrystals that possess a magnetic moment that is large enough to allow for spatial guidance, by using relatively weak external magnetic fields. Conversely, the iron oxide nanoparticles that are typically smaller than 20 nm, and therefore in superparamagnetic regime, do not generate a sufficient magnetic force for their effective spatial guidance in suspension.

However, the main advantage of having superparamagnetic nanoparticles is the simplicity to prepare stable colloidal suspensions, because superparamagnetic nanoparticles do not feel each other magnetically at physiological conditions (i.e., at room temperature and Earth’s magnetic field), and hence behave as “non-magnetic” nanoparticles. On the contrary, ferrimagnetic nanoparticles behave as tiny permanent magnets, due to their remanent magnetization. Although there are a few studies where chain-like assemblies of ferri/ferromagnetic nanoparticles were demonstrated, these assemblies’ morphologies are poorly controlled, due to the spontaneous dipole-induced magnetic interactions among nanoparticles.

**Figure 6:**
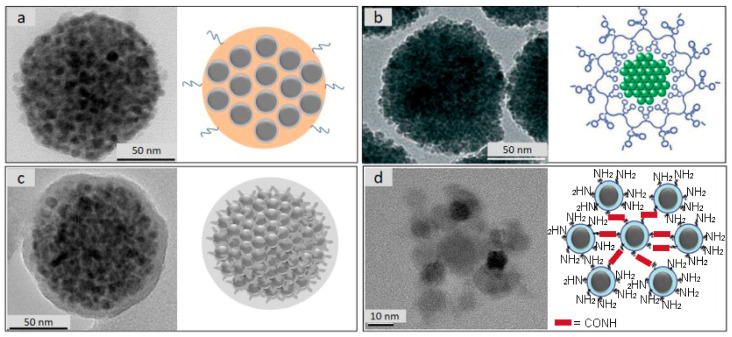
TEM images and corresponding schematic illustrations of synthesized magnetically guidable nanoparticle clusters. (**a**) Composite magnetic nanoparticle cluster composed of superparamagnetic maghemite nanoparticles and biocompatible polymer [85] (**b**) One-pot solvothermal synthesis of clusters composed of many superparamagnetic magnetite nanoparticles. Adapted with permission from [84] Copyright 2007, with permission from Wiley. (**c**) Emulsion/evaporation-based clustering of hydrophobic maghemite nanoparticles following silica coating. Reprinted from [29], Copyright 2014, with permission from Elsevier. (**d**) Chemical cross-linking method of differently functionalized superparamagnetic maghemite nanoparticles forming synthesized magnetically guidable nanoparticle cluster, adapted from [42]. CC BY 4.0 license.

The main disadvantage of using superparamagnetic nanoparticles is their inability to be magnetically guided, due to the insufficient magnetic force that is generated, even when they are exposed to magnetic fields with the highest gradients. However, one possible solution to this issue is based on the clustering of many superparamagnetic nanocrystals into larger nanoparticles that preserve the superparamagnetic properties, while possessing enhanced magnetic moment that is suitable for effective spatial guidance of the particles in suspension. Therefore, different approaches of superparamagnetic nanoparticle clustering have been proposed in the last two decades, including: one-pot nanoparticle cluster synthesis method, solvothermal methods, chemical cross-linking of nanoparticles in the cluster, preparation of composite nanoparticle clusters with polymers, and emulsion/evaporation-based clustering of hydrophobic nanoparticles, among other strategies (Figure 6) [29,41,42,75,76,77,78,79,80,81,82,83,84,85,86,87]. Krasia-Christoforou et al. has recently presented an elegant topical review on magnetic nanoparticle clustering that is available for further reading [88].

#### 2.2.2. Magnetic Field-Assisted Particles’ Assembly into Nanochains

Magnetic assembly is considered unique for structuring magnetically guidable particles into more complex hierarchical architectures. The most typical example is the formation of 1D nanochains that are composed of permanently linked spherical nanoparticles [73]. The nanoparticles need to be able to spatially move in the presence of magnetic field gradients, for the exploitation of the magnetic field in the assembly process. However, this is not possible with the individual superparamagnetic nanoparticles in stable colloidal suspension, as explained in the previous section. Conversely, the multicore clusters of superparamagnetic nanoparticles are able to spatially move, hence form chain-like formations, in moderate magnetic fields. Nevertheless, there are examples of magnetic chains that are formed from single-domain particles, for instance, cobalt ferrite nanoparticles, where 1D alignment was obtained with magnetic-field assistance [89]. Indeed, the assembled particles were simultaneously linked by exploiting Diels–Alder cycloaddition to preserve anisotropic assembly. Interestingly, the covalent bonds that were formed via Diels–Alder reaction were thermoreversible, thus allowing for on-demand chains’ disassembly through heat exposure, or by using remotely controlled magnetic fluid hyperthermia. As demonstrated in this study, the chains produced were not well-defined, rather highly curved and of heterogeneous lengths. The reason lays in the fact that these individual particles behave as tiny permanent magnets. Clearly, such nanoparticles are probably impossible to assemble in a fully controllable way, due to spontaneous dipole interactions among nanoparticles that disturb the externally triggered magnetic assembly process [90].

An interesting method of magnetic structuring was presented by Bannwarth et al. where SPION-loaded polymeric particles were magnetically assembled into different morphologies, while the assembled structures were fixated through the formation of permanent linkages between neighboring particles, using thermal sintering of the polymeric component [91]. Here, superparamagnetic particles exposed to the magnetic field generated sufficient attractive magnetic force to overcome the energy barrier created by the highly negative zeta potential of the particles (from −40 mV to −60 mV) and resulting in electrostatic repulsive forces between adjacent particles. The simultaneous magnetic assembly and thermal treatment enabled the transformation of chain-like morphology into smooth magnetic fibers [92]. Interestingly, continuous magnetic fibers can also be fabricated without magnetic field assistance, for instance by mixing magnetic nanoparticles and polymers through electrospinning, as our group has recently demonstrated [93].

In general, the magnetic field-assisted nanoparticles’ assembly using superparamagnetic multicore nanoparticle clusters enables the greatest level of control over the shape and dimensions of hierarchical magnetic 1D structures [28,94,95,96,97]. The pioneering work of Yadong Yin demonstrated the synthesis of magnetically responsive photonic nanochains, so that monodispersed nanoparticle clusters were magnetically aligned, and the chain-like formations were precisely fixated within a silica shell [95]. The authors showed that the timing and duration of magnetic exposure—from about 0.5 s to about 4 s—can be used to control the length of the nanochains in the range between two and ten micrometers, respectively. Another study by Zhou et al. applied mussel-inspired polydopamine (PDA) coating as fixating agent for the magnetically aligned structures [98]. Here, the control over the length of the nanochains was determined by the duration of the ultrasound exposure, just before the fixation of aligned structures with a PDA shell. The sonication of the reaction mixture—for 10 s or 3 s—before it was left undisturbed for PDA deposition, resulted in the average length of nanochains corresponding to 1.0 or 2.7 micrometers, respectively. The authors suggested that sonication reduced the length of aligned nanoparticles before the structure was fixated by PDA. The same group demonstrated another application where similar nanochains fixated with PDA and were used as stirring bars for rapid liquid mixing in a microfluidic device [96]. Here, the authors demonstrated that the length of the nanochains can be reduced after their synthesis and coating with PDA, through the application of 3 s sonication pulses. Indeed, these nanochains were fragile and could easily break into shorter fragments of 3 μm from the originally synthesized nanochains with an average length of 10 to 20 μm. Recently, Deng’s group demonstrated the facile magnetic-field assisted approach for the synthesis of magnetic nanochains composed of solvo-thermally synthesized nanoparticle clusters that were previously developed by Yin’s group [94]. These nanochains were further coated with an additional layer of mesoporous silica with radially aligned pores, which could be used for drug or nanocatalyst loading. The authors declared that nanochain lengths could be tuned in the range between 1 and 15 µm.

**Figure 7:**
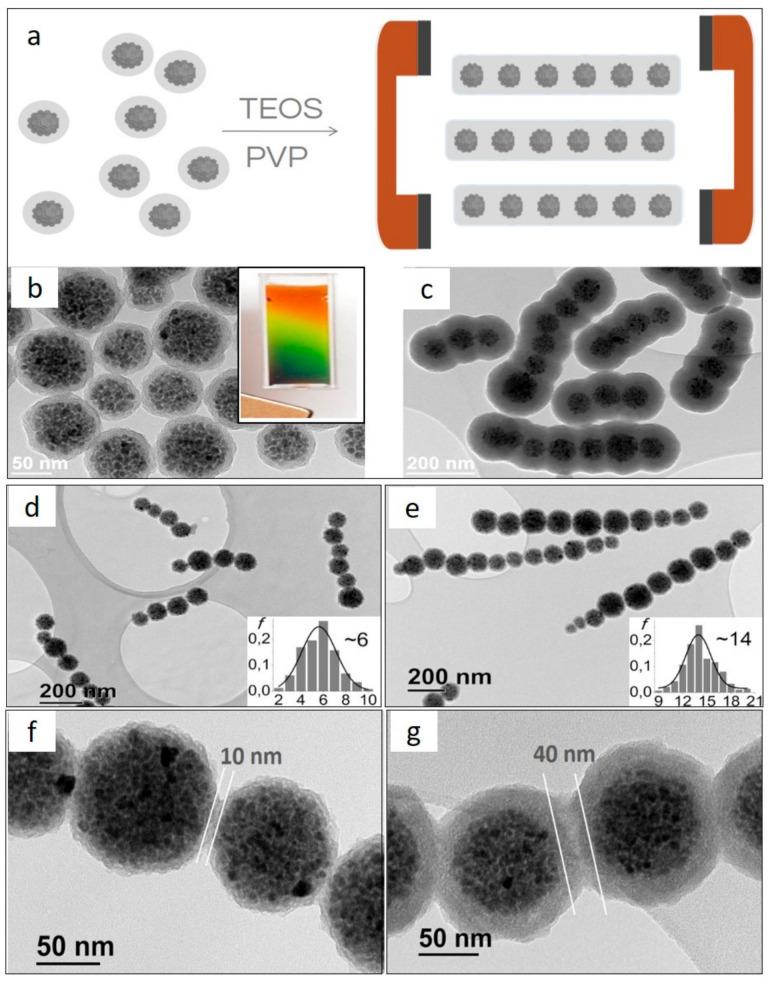
Schematic illustration and corresponding TEM images of key steps in the magnetic nanochain formation. (**a**) Alignment of superparamagnetic maghemite nanoparticle clusters in dynamic magnetic field. (**b**) Nanochains’ building blocks consisting of silica-coated nanoparticle clusters. Inset: the resulting suspension is a magnetically tunable photonic crystalline liquid resulting in a structural color when the suspension is exposed to a weak external magnetic field. (**c**) Rigid magnetic nanochains coated with fixating silica shell. (**d**) Short nanochains composed of ca. 6 clusters (length ca. 400–700 nm). (**e**) Magnetic nanochains composed of ca. 14 clusters (length ca. < 1 µm). (**f**) Short inter-particle distance between adjacent particles in the nanochain by choosing building blocks with thin primary silica shell of 5 nm. (**g**) Large inter-particle distance between adjacent particles in the nanochain by choosing building blocks with thick primary silica shell of 20 nm. Reprinted with permission from [28], Copyright © 2021, with pemission from American Chemical Society.

As demonstrated in the last paragraph, different research groups are able to synthesize superparamagnetic nanochains composed of nanoparticle clusters with outstanding properties, although the nanochain lengths always exceed 1 µm. This feature seriously limits their potential use in vivo for medicine, because it is broadly accepted that an ideal length should be in the nanoscale range. Our group has demonstrated a facile and dynamic approach for the synthesis of nanochains with submicron-scale length (Figure 7) [28]. We showed a versatile and flexible approach based on the simultaneous magnetic assembly and stirring-induced disassembly of nanoparticle clusters into nanochains of well-defined lengths. The magnetic-field induced assembly of nanochains was temporarily stabilized with bioinspired polyvinylpirrolidone (PVP) backbone and fixated by the deposition of an additional silica layer. This innovative approach enabled an excellent length control for the short nanochains composed of less than 20 nanoparticle clusters (Figure 7c–e). It was demonstrated that the nanochain length could be controlled, thanks to a fine interplay among different synthetic parameters, such as the time for which the suspension was exposed to a magnetic field, the PVP concentration, the magnitude of the magnetic field, the clusters’ volume fraction, the stirring rate, and the magnetic-field exposure time. Notably, this approach allows also for the fine-tuning of the spacing between adjacent particles inside individual nanochains, through the simple control over the thickness of the primary silica shell on the assembled particles (Figure 7f,g). It is worth noting that the initial nanoparticle clusters need to form a highly stable colloidal suspension, in order to obtain such control over all the crucial synthetic parameters. Interestingly, the suspension of such nanoparticle clusters is basically the magnetically tunable photonic crystalline liquid, which gives rise to a structural color when exposed to the weak external magnetic field (insertion in Figure 7b) [99].

#### 2.2.3. Polymer-Assisted Particles’ Assembly into Nanochains

One of the first approaches for the synthesis of magnetic nanochains that are composed of individual nanoparticles is based on using dextran [100]. It was shown that high-molecular-weight polymer molecules, with the functional groups distributed along the backbone, strongly associate with iron oxide nanoparticles. This affinity drives the attachment of nanoparticles to the backbone, which results in the formation of magnetic 1D assemblies [37]. The anisotropic assembly is composed of 5-nm-sized nanoparticles aligned as a string, with an overall length of 50 nm or 100 nm, when 20 kDa dextran or 40 kDa were used, respectively. Then, similar nanochains were used as drug delivery systems to co-deliver chlorotoxin and curcumin for lung anticancer therapy [101].

Recently, another study by Zhao et al. demonstrated the use of similar dextran-supported iron oxide nanochains for synergistic MRI-guided photothermal and magneto-mechanical destruction of tumor cells [40]. Zhou et al. demonstrated a one-pot synthesis approach to obtain nanochains that display magnetic particles cross-linked by biocompatible poly(cyclotriphosphazene-co-4,4′-sulfonyldiphenol) polymer [102]. However, the authors have not demonstrated the ability to control the length of spontaneously assembled nanochains. Similarly, the same concept has been applied by Yang et al., although with limited control over the length of the so-formed nano-sized stirring bars [103].

The idea of using polymers that associate with the inorganic nanoparticles is potentially applicable to other types of versatile inorganic nanoparticles, for instance, based on gold, so that thiol-decorated polymers can guide the formation of hierarchical assemblies [104]. For instance, Lee et al. demonstrated a universal mussel-inspired polymeric template that can assemble a wide variety of nanoparticles into 1D assemblies including gold nanoparticles and quantum dots [105].

#### 2.2.4. Colloidal-Assisted Particles’ Assembly into Nanochains and 1D Arrays

**Figure 8:**
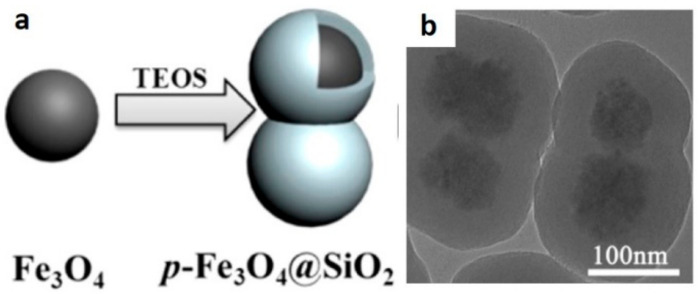
Schematic illustration of the fabrication process of short peapod-like magnetic nanochains. (**a**) Schematic synthesis protocol and (**b**) TEM image of the resulting nanochains. Reprinted with permission from [106], Copyright © 2021, with pemission from American Chemical Society.

Anisotropic peapod-like assemblies can be achieved through a gentle adjustment of the stability of colloidal suspensions. Wang et al. showed that multicore nanoparticle clusters self-assemble into very short nanochains composed of mainly two clusters without the assistance of any template or magnetic field (Figure 8) [106]. The assembled structure is fixated with the deposition of a silica shell. Interestingly, a slightly larger addition of the silica precursor, i.e., tetraethoxysilane (TEOS), promoted the spontaneous assembly of peapod-like structures. Conversely, just a slightly smaller addition of TEOS resulted in the coating of individual nanoparticle clusters. Although the authors have not fully clarified the mechanisms involved in this particular assembly process, the extent of hydrolyzed silane species might impair the colloidal stability. At the same time, it could also speed up the condensation of silica that is supposed to limit the collisions among particles. As a result, in the majority of cases, two particles were assembled in these short peapod-like nanochains.

**Figure 9:**
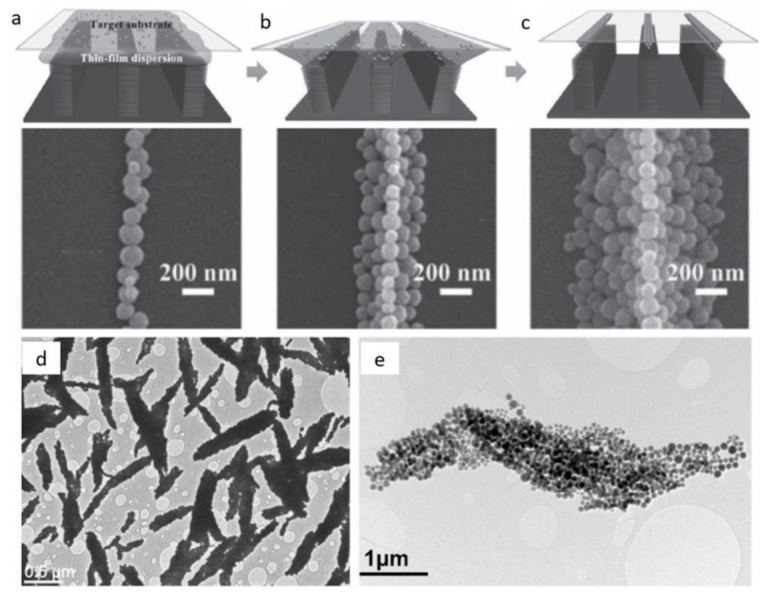
(**a**–**c**) Schematic illustration of a method for fabricating striped patterns of magnetic nanoparticles. (**a**) Template consisting of elongated ridges and gaps, and a flat target substrate. (**b**) After suspension deposition, water is evaporated and the film is divided in the sections along the ridges of the template. (**c**) 1D arrangement of the nanoparticles. Reproduced with permission from [107], Copyright 2016, with permission from Wiley. (**d**,**e**) Colloidal approach for the magnetic structuring in suspension. Reproduced with permission from [28], Copyright © 2021, with pemission from American Chemical Society. (**d**) Low-magnification TEM image of anisotropic magnetic nanobundles composed of superparamagnetic nanoparticle clusters, and (**e**) TEM image of one magnetic nanobundle.

The capillary-bridge-mediated assembly (CBMA) is a kind of colloidal process that enables the assembly of superparamagnetic magnetite nanoparticles into 1D arrays with a high aspect ratio [107]. The method is based on asymmetric wettability of a micro-structured template, thus enabling capillary bridges with 1D configuration of assembled nanoparticles (Figure 9a–c). These high-aspect-ratio 1D arrays possess a high level of anisotropy, resulting in the biomimicry of magnetic field perception. However, the main drawback of the presented approach is a difficult scale-up, because these 1D arrays require a peculiar template that involves many complicated engineering steps. In the future, novel colloidal processing approaches are needed to fabricate superparamagnetic anisotropic structures on a large scale. In this regard, our group showed progress in this direction through the precise colloidal processing of superparamagnetic nanoparticles, thus enabling the synthesis of micro-sized superparamagnetic bundles on a large scale and without any template (Figure 9d,e) [28]. We only exploited the interplay between colloidal properties in suspension and simple magnetic-field assistance without the use of a template, for the structuring of anisotropic micron-sized particles starting from nanoscale building blocks.

#### 2.2.5. Solid Support-Assisted Particles’ Assembly into Nanochains

**Figure 10:**
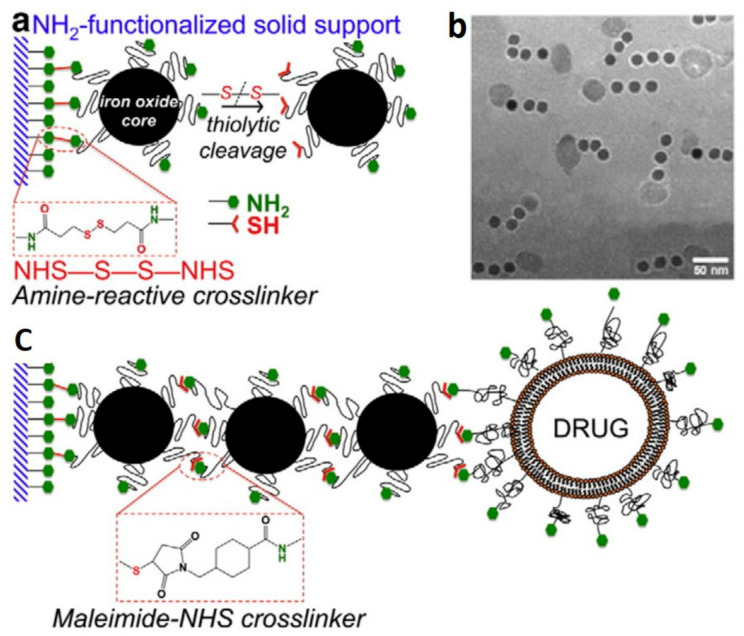
Reaction scheme of the controlled assembly of magnetic nanochains using solid-phase chemistry. (**a**) Precise control over the chemical functionality of amines and thiols on the surface of individual magnetic nanoparticles. (**b**) TEM image of fabricated nanochains, each one composed of three units of nanoparticles. (**c**) Schematic illustration of a multi-step process to produce nanochains with a terminally bonded liposome drug carrier. Reproduced from [110], Copyright 2013, with permission from Elsevier.

An alternative approach to enable the synthesis of magnetic nanochains that are composed of individual superparamagnetic nanoparticles is based on using solid supports (Figure 10). Asymmetric surface chemistry could be achieved by using acrylate resins functionalized with amino groups. Then, the resins were modified with an amine-reactive, homo-bifunctional, and cleavable, cross-linker that was bound to amino-functionalized magnetic nanoparticles [108]. Due to steric hindrance, the magnetic nanoparticles could react with the resin through just a small portion of their surface area, thus becoming Janus nanoparticles. Such bifunctional magnetic nanoparticles could guide further asymmetric spatial positioning of chemical functionalization. This was shown through subsequent addition of reactive magnetic nanoparticles. Overall, the authors performed this reaction twice, and hence they generated nanochains, each one composed of three units of magnetic nanoparticles. Finally, the nanochains were released from the solid support after thiolitic cleavage using dithiothreitol [109]. Although the nanochains showed promising results in cancer therapy due to their ability to carry potent chemotherapeutics, the main disadvantage of the presented approach remains the scalability of such nanochains’ production [110,111]. However, the approach is elegant and allows for the synthesis of nanochains of any type of non-magnetic nanoparticle with proper functional groups as building blocks, because the assembly is not magnetically assisted and does not require magnetically guidable nanoparticles.

## 3. Biomedical Applications of Magnetic Nanochains

Magnetic nanochains show great potential as a novel example of anisotropic nanostructures that can be guided remotely through the application of external magnetic fields [28,112,113,114]. In particular, their morphological anisotropy opens new possibilities to exploit magneto-mechanical effects in the treatment of diverse diseases, because nanochains can follow the rotational movement of rotating magnetic field at low frequencies (up to hundreds of Hz), contrarily to individual spherical nanoparticles [115,116]. Such a movement of rigid nanochains can transduce the magnetic force into mechanical torque exerting on a nearby targeted tissue [36]. Alternatively, their rotational movement can accelerate drug release from the nanochain surface on demand, and this represents a radically new means of controlled drug release [94]. However, there are not many examples where intact magnetosome chains were used in biomedical applications, because individual ferrimagnetic magnetite particles are usually separated from the magnetotactic bacteria during the harvesting process [117,118]. Therefore, mainly bioinspired artificial nanochains demonstrated their potential in diverse biomedical applications. In this section, we focus on magnetic nanochains’ assemblies, and not on spherical magnetic nanoparticles for which there is abundant literature available.

Alphandery et al. demonstrated the use of magnetic nanochains, which were extracted from magnetotactic bacteria, for anticancer therapy through exposure to an alternating magnetic field of frequency 183 kHz and field strengths between 20 and 60 mT [119]. The authors showed complete eradication of the tumor under the skin of a mouse. The magnetic fluid hyperthermia effect of nanochains was compared to individual magnetosome particles, thus confirming the chains’ superior efficiency. This observation can be ascribed to uniform heating distribution within the tumor tissue generated by the chains of the magnetosomes, compared to the individual magnetosomes.

**Figure 11:**
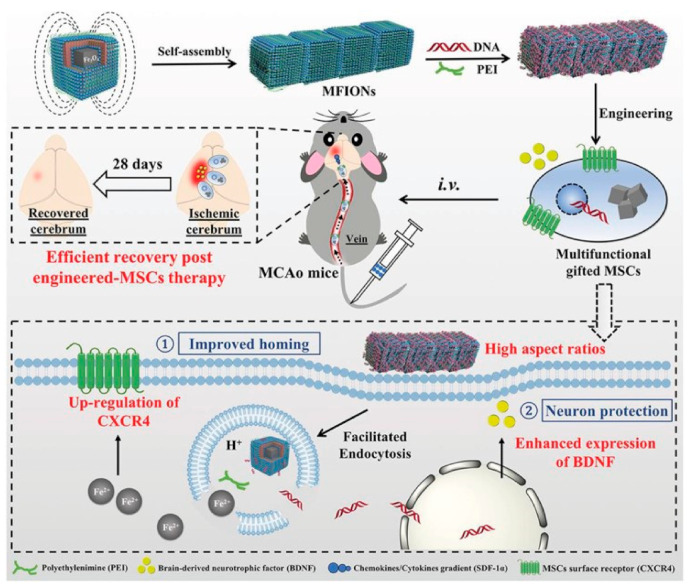
Schematic illustration of ferrimagnetic nanochain assembly, their surface modification, transfection of mesenchymal stem cells, and in vivo application to trigger post-stroke recovery in a mouse model. Reproduced with permission from [26], Copyright 2019, with permission from Wiley.

Recently, Zhang et al. have developed ferrimagnetic nanochains that were composed of magnetosome-like uniform ferrimagnetic nanocubes (Figure 11) [26]. The chains were formed spontaneously, due to attractive dipole interactions. As a result, the length of such nanochains was difficult to control. Nevertheless, these nanochains were coated with polyethylene amine and conjugated with pDNA, for use as gene-transfecting agents for mesenchymal stem cells (MSCs) towards highly efficient post-stroke recovery. The nanochains were internalized by MSCs, and they triggered the overexpression of brain-derived neurotropic factors to allow for the treatment of ischemic cerebrum. This study demonstrated a simple and effective approach to genetically engineer specific cells using magnetic chain-like particles. Finally, the authors demonstrated the recovery of brain functions and a significant reduction in post-stroke mortality comparable to those attained with the more traditional viral transfection of cells.

**Figure 12:**
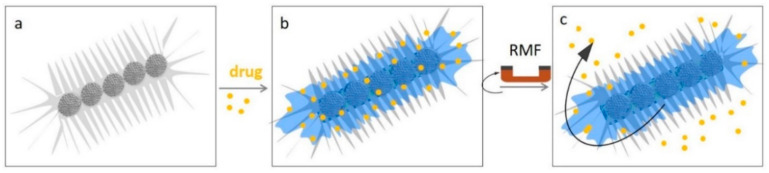
Future application of multifunctional short nanochains carrying drug molecules. (**a**) Schematic representation of short nanochain with radially aligned pores on the surface, whose design and production has recently been described [39]. (**b**) Schematic representation of drug-loaded nanochains. (**c**) Drug release can be triggered through exposure to a rotating magnetic field (RMF). Reproduced from [39] under a Creative Commons license.

Our group has demonstrated the biocompatibility of bioinspired short nanochains, which were composed of approximately 5 nanoparticle clusters (with overall length < 1 µm), and that were fixated with a rigid silica shell [36]. We showed that these nanochains were internalized in cancer cells in 2D and 3D culture models, that they were non-toxic, and that they were able to eradicate cancer cells and disrupt the extracellular matrix. Indeed, after near-infrared-light irradiation (with a wavelength of 808 nm) the nanochains became efficient cytotoxic photothermal agents, as they effectively eradicated tumor cells in vitro. Additionally, the nanochains also locally overheated the extracellular collagen matrix that was self-secreted by engineered cell sheets. Interestingly, the tumor microenvironment is tightly cross-linked with a collagen matrix, which reduces the capability of conventional drugs’ penetration deeply into the tumor. Therefore, the magnetic nanochains with their ability to disrupt the collagen matrix have a significant therapeutic value. In the future, such short nanochains can be used as multifunctional agents, to carry therapeutic molecules while generating heat locally (Figure 12).

Wan et al. have recently presented relatively long magnetic nanochains with lengths between 1 µm and 15 µm that were rigidified with a thin and robust silica shell [94]. The authors coated them with an additional mesoporous silica shell, using interfacial co-assembly. The pores in the silica shell allowed for efficient drug loading and controlled release in vitro. In particular, the zoledronate-loaded nanochains were sensitive to rotating magnetic field exposure and suppressed the osteoclasts’ differentiation of bone-marrow-derived macrophages. However, the control sample of individual spherical nanoparticle clusters showed no response to the magnetic field, due to the lack of shearing force. They confirmed that the shearing force of rotating, anisotropic nanochains induced sufficient effects in cells to attain the desired outcome.

Yang et al. demonstrated a one-pot polymer-assisted formation of nanochains with lengths between 60 and 110 nm, although with relatively poor control over the nanochains’ shape and length, as evidenced from the electron microscopy images [101]. The nanochains were loaded with chlorotoxin and curcumin, and then lung cancer cells were targeted and their growth was effectively inhibited. In vivo targeting was monitored by magnetic resonance imaging and fluorescence imaging, whereby the small tumor was detected by enhanced image contrast. These nanochains are thus considered promising for the early diagnosis and therapy of lung tumors.

**Figure 13:**
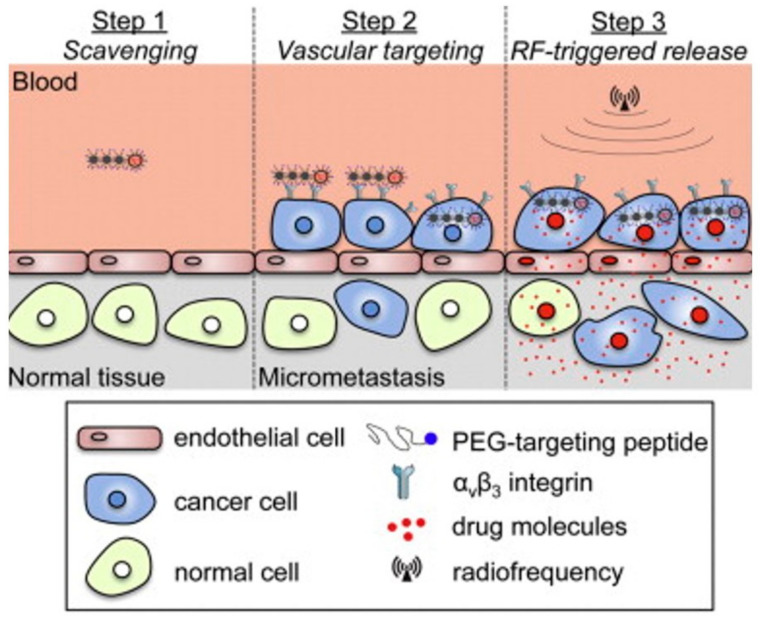
Schematic presentation of therapeutic anticancer approach based on nanochains following three steps. (1) Short nanochain loaded with drug circulates in the blood. (2) Nanochains’ target micrometastases. (3) Radiofrequency-triggered drug release from the nanochains. Reproduced from [110] Copyright 2013, with permission from Elsevier.

Peiris et al. have shown an approach to treat cancer micrometastases, using short and well-defined magnetic nanochains [110]. Micrometastases are extremely difficult to treat because they are nearly inaccessible to drugs, since they are small in size, and highly dispersed in different organs. The authors prepared small and flexible nanochains with the ability to target and persist at micrometastatic sites (Figure 13). Moreover, these nanochains were designed as carriers, sensitive to radiofrequency pulses. These features promoted the effective eradication of tumor cells, using a low dose of a cytotoxic drug.

There is a significant pool of publications related to individual magnetosome nanoparticles and their uses in the biomedical field which were not a focus of our review. For instance, the magnetosome particles have been used in magnetic resonance imaging, magnetic hyperthermia, and drug delivery [27,120,121,122,123,124,125,126]. Finally, we recommend existing review papers related to magnetosome particles for further reading [17,127,128,129,130,131].

## 4. Future Prospects and Conclusions

Despite the enormous research progresses that has enabled new discoveries and an improved understanding of basic phenomena in physics, materials science, and life sciences in the last century, some life forms—as simple as prokaryotic single cells, such as magnetotactic bacteria—have not yet been completely understood [34]. Evolution and organisms’ adaptation to an ever-changing world are resulting in the careful selection of the forms, materials, and environments to increase the chance for long-term survival. Magnetotactic bacteria are no exception and, most probably, their magnetosomes mineralize iron oxide crystals in the form of magnetite, because it has the largest magnetic moment per volume among stable iron oxides. Furthermore, magnetite nanoparticles in magnetosomes are single domain, hence ferromagnetic, which maximizes the efficiency of their magnetic sensing. The magnetosome spatial location inside bacterial cells is carefully defined even before the magnetite crystals become fully mature. Every single crystal is encased within a lipid bilayer membrane, which is abundant with specific functional proteins, to yield magnetosomes. The organic shell is very important for the fine regulation of magnetite biomineralization and magnetosome attachment to the fibrillar skeleton, which aligns magnetosomes into 1D chains. Furthermore, the magnetosome’s organic shell thickness regulates the distance between adjacent ferrimagnetic particles, and hence finely tunes attractive magnetic dipole interactions between magnetite nanocrystals inside a magnetosome chain. The aligned magnetosome chain structure can withstand a magnetic force of 25 pN and magnetic field strength of 30 mT [25]. These values are orders of magnitude larger than the ones that bacteria are exposed to at the Earth’s surface. It would thus appear that bacteria developed the optimal nanoarchitecture for their needs. Researchers have long tried to guide biomineralization by feeding bacteria with salt of magnetic ions, and by manipulating their genetic machinery [121,132,133]. The overall aims of these efforts are to gain a better understanding of the mechanisms involved in the biomineralization process, as well as the production of artificial nanochains on a large scale in the lab.

There are just a few examples of intact magnetosome chains being used for biomedical applications [119]. One of the reasons may be the challenging separation and harvesting of nanochains from bacteria without structural damage, since the procedure typically requires the use of chemicals (such as surfactants) and mechanical treatments (such as exposure to ultrasonic probes). Indeed, the process can easily be harsh enough to disrupt the fibrillar network that supports the magnetosomes’ chains, as well as, at least partially, destroy the sensitive magnetosome membrane [134,135]. Therefore, as evidenced from the electron microscopy analyses in the majority of publications, the results of nanochain harvesting are usually isolated magnetite nanocrystals rather than intact magnetic nanochains. Regardless of the result of nanochain harvesting, the obtained magnetite nanoparticles in the individual or chain-like form have a strong tendency to magnetically agglomerate, due to spontaneous attractive magnetic dipole interactions among ferrimagnetic nanoparticles. The magnetic dipole interactions among ferrimagnetic particles depend on the inter-particle distance, and are hence significantly increased if particles reach close proximity. This phenomenon constitutes a real challenge to preserve magnetic nanochains of natural origin during their isolation. Conversely, this issue is not normally encountered during the existence of the bacterium, because two bacteria can never come in such close proximity to magnetically attract each other so intensely to damage their 1D chain-like assembly.

In conclusion, we foresee two general directions to follow in order to fully exploit the potential of magnetic nanochains in biomedical applications. First, additional efforts should be directed in the development of new procedures to enable the efficient isolation of magnetosome chains that preserve both the 1D assemblies and magnetosome membranes. This is crucial for the exploitation of the anisotropic nanostructures in advanced magneto-mechanical treatments, for which the individual ferrimagnetic nanoparticles are useless. Indeed, removal of the magnetosome membrane from the ferrimagnetic particle surface might impair their colloidal stability in an irreversible manner. Secondly, novel assembly approaches to obtain bioinspired magnetic nanochains are highly desirable. We believe that special efforts are needed in the design of novel magnetosome-like nanoparticles to overcome some issues the researchers face with isolated magnetosome crystals. Additionally, the formation of bioinspired chains of magnetic nanoparticles should allow for (1) the precise tailoring of nanochain dimensions, (2) a wide choice over suitable types of materials to connect magnetic nanoparticles into rigid or flexible 1D assemblies, and (3) ease of production scale-up. Finally, we conclude by noting that magnetotactic bacteria apparently created their magnetic assemblies, which represent a great evolutionary achievement, for their exclusive use, since these nanochains’ exploitation by humans demands a lot of effort and innovative solutions. Clearly, much more progress is needed to address all the challenges discussed in this review before we will be able to fully benefit from these fascinating anisotropic nanostructures and their magnetic properties in medicine.

## Figures and Tables

**Table 1:** Characterization techniques for magnetic nanostructures evaluation.

Type of Nanostructure	Characterization Technique	Refs.
Magnetosome chains, magnetosome particles	X-ray diffraction	[25,26,27]
Magnetosomes, magnetosome chains, short nanochains, magnetic nanobundles, bioinspired nanoparticle clusters	Transmission electron microscopy (TEM)	[25,28,29,30,31,32,33,34,35]
Magnetosome chains	Optical microscopy	[25,32]
Bioinspired short nanochains, magnetic nanobundles, bioinspired nanoparticle clusters	Scanning electron microscopy (VSM)	[28,29,36,37,38]
Bioinspired nanoparticle clusters	Mössbauer spectroscopy	[29]
Magnetosome chains	Cryo-electron tomography	[32]
Bioinspired short nanochains	Zeta potential measurement	[36]
Bioinspired short nanochains, bioinspired nanoparticle clusters	Brunauer–Emmett–Teller analysis (BET)	[39]
Bioisnpired chains, nanoparticle clusters	Thermogravimetric analysis (TGA)	[40,41]
Nanoparticle clusters	Dynamic light scattering (DLS)	[41,42]
Magnetosomes	Ferromagnetic resonance spectroscopy	[43]

## Data Availability

The data presented in this study are available on request from the corresponding author.
